# Pan-genomic analysis to redefine species and subspecies based on quantum discontinuous variation: the *Klebsiella* paradigm

**DOI:** 10.1186/s13062-015-0085-2

**Published:** 2015-09-30

**Authors:** Aurélia Caputo, Vicky Merhej, Kalliopi Georgiades, Pierre-Edouard Fournier, Olivier Croce, Catherine Robert, Didier Raoult

**Affiliations:** URMITE, UMR CNRS 7278-IRD 198, Faculté de Médecine, Aix-Marseille Université, 27 Boulevard Jean Moulin, 13385 Marseille, Cedex 5 France; Departement of Biological Sciences, University of Cyprus, P.O. Box 20537–1678, Nicosia Cyprus, Greece

**Keywords:** Pan-genome, *Klebsiella pneumoniae*, Taxonomy

## Abstract

**Background:**

Various methods are currently used to define species and are based on the phylogenetic marker 16S ribosomal RNA gene sequence, DNA-DNA hybridization and DNA GC content. However, these are restricted genetic tools and showed significant limitations.

**Results:**

In this work, we describe an alternative method to build taxonomy by analyzing the pan-genome composition of different species of the *Klebsiella* genus. *Klebsiella* species are Gram-negative bacilli belonging to the large *Enterobacteriaceae* family. Interestingly, when comparing the core/pan-genome ratio; we found a clear discontinuous variation that can define a new species.

**Conclusions:**

Using this pan-genomic approach, we showed that *Klebsiella pneumoniae* subsp. *ozaenae* and *Klebsiella pneumoniae* subsp. *rhinoscleromatis* are species of the *Klebsiella* genus, rather than subspecies of *Klebsiella pneumoniae*. This pan-genomic analysis, helped to develop a new tool for defining species introducing a quantic perspective for taxonomy.

**Reviewers:**

This article was reviewed by William Martin, Pierre Pontarotti and Pere Puigbo (nominated by Dr Yuri Wolf).

**Electronic supplementary material:**

The online version of this article (doi:10.1186/s13062-015-0085-2) contains supplementary material, which is available to authorized users.

## Definitions

**Table:** 

Term	Definitions
Accessory genome	Set of genes present in more than one strain but not in all strains studied
Core genome	Genes present in all strains studied
Pan-genome	Gene pool present in the genomes of a group of organisms
Species	Homogeneous group of isolates characterized by many common features

## Background

**Fig. 1:**
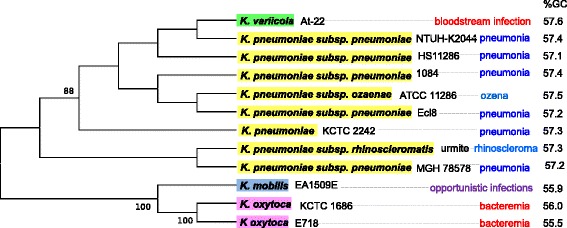
A 16S rRNA-based phylogenetic tree of all strains studied with their associated pathotype and GC %

Taxonomy is essential for the identification, nomenclature and classification of bacterial species. Bacterial taxonomy has undergone many changes since the first attempts to establish a bacterial classification [1]. Pathogenic bacteria were initially classified as distinct species according to their pathotype. In this study, we took the *Klebsiella* species as model. The genus *Klebsiella* consists of organisms that are usually non-motile, with the exception of *Klebsiella mobilis* (considered as ‘*Enterobacter aerogenes*’ because of this mobility) [2] and Gram-negative rods. Species of the genus *Klebsiella* are important common pathogens causing variable clinical syndromes including nosocomial infections for *Klebsiella mobilis*, bloodstream infections and bacteremia for *Klebsiella variicola* and *Klebsiella oxytoca*. Three closely-related species, *Klebsiella pneumoniae*, *Klebsiella rhinoscleromatis* and *Klebsiella ozaenae* have been identified as pathovars because they cause distinguishable diseases of the respiratory tract: *K. pneumoniae* is responsible for the majority of human *Klebsiella* infections [3], causing pneumonia. *K. ozaenae* is rarer and is found in chronic diseases of the respiratory tract, especially atrophic rhinitis (ozena); it can also be isolated from the sputum, urine and, exceptionally, from blood cultures. *K. rhinoscleromatis* causes rhinoscleroma (a tumor of the nose) (Fig. 1). The metabolic activities of these three species *in vitro* also differ. Thus, the fermentation of dulcitol and sorbose and the catabolism of d-tartrate and, secondly, the fermentation of rhamnose and adonitol, were additional criteria used to define the three biovars [4].

Over time, the taxonomy of bacteria has been reorganized based on a combination of phenotypic and genotypic properties [5]. The genotypic criteria by which bacterial species were first characterized included the genomic GC content composition. Later, DNA-DNA hybridization experiments were used for comparisons with the closest phylogenetic neighbors [6]. In the 1990s, the sequencing of the 16S rRNA gene led to a revolution in the classification of bacterial species [7], enabling the re-classification of living organisms [8]. Currently, a threshold identity of 98.7 % in the 16S rRNA sequence is used to define a new bacterial species [9–11]. Thus, the taxonomic study of the *Klebsiella* genus, based on 16S rDNA and DNA-DNA hybridization, reclassified *K. ozaenae* and *K. rhinoscleromatis* as subspecies of *K. pneumonia* (Fig. 1).

Recently, improvements in genome sequencing have facilitated the study of bacterial species, particularly by analyzing their taxonomy [2, 12]. Previous studies demonstrated the importance of genomics for bacterial taxonomy by assessing the presence of indels or single nucleotide polymorphisms (SNPs) in conserved genes [13], comparing orthologous genes [7] and studying metabolic pathways [14]. In this work, we developed another method to build a taxonomy that takes advantage of genome analysis and pan-genome definition [15]. Indeed, the comparison of the core/pan-genome ratios of the different *Klebsiella* species revealed that *K. pneumoniae* subsp. *ozaenae* and *K. pneumoniae* subsp. *rhinoscleromatis* exhibit many differences between themselves as well as with *bona fide Klebsiella* species. This finding supports the claim that *K. pneumoniae* subsp. *ozaenae* and *K. pneumoniae* subsp. *rhinoscleromatis* are distinct species of the *Klebsiella* genus. This work introduces pan-genome analysis as a novel tool to define species and represents a great leap forward in bacterial taxonomy.

## Methods

### Genome sequencing and annotation

Genomes from *K. pneumoniae* subsp. *ozaenae* and *K. pneumoniae* subsp. r*hinoscleromatis* were sequenced using shotgun sequencing method with IonTorrent_Lifetechnologies and the Roche_454 method. For IonTorrent sequencing, genomic DNA was mechanically fragmented in Covaris microTubes to generate a fragment size distribution from 180 to 220 bp and purified through Ampure Beads (Agencourt, Beckman). The fragmented library was constructed using adaptor ligation according to the manufacturer's instructions (Life Technologies). Template preparation, emulsion PCR and Ion Sphere Particle (ISP) enrichment was performed using the Ion One Touch kit (Life Technologies). The quality of the resulting ISPs was assessed using a Qubit 2.0 Fluorometer (Life Technologies), and samples were loaded twice and sequenced on a 316 chip (Life Technologies). Finally, 3,567,359 reads for *K. pneumoniae* subsp. *ozaenae* and 3,325,174 reads for *K. pneumoniae* subsp. *rhinoscleromatis* were generated. A 5 kb paired end library was constructed with 5 μg of DNA according to the 454_Titanium paired end protocol and to the manufacturer’s instructions. This was mechanically fragmented using the Covaris device (KBioScience-LGC Genomics, Queens Road, Teddington, Middlesex, TW11 0LY, UK) with miniTUBE-Red 5Kb. DNA fragmentation was viewed using the Agilent 2100 BioAnalyzer on a DNA labchip 7500 with an optimal size of 4.9 kb. Circularization and nebulization were performed on 100 ng of the sample. After PCR amplification through 17 cycles followed by a double size selection, the single-stranded paired end library was then loaded onto a DNA labchip RNA pico 6000 on the BioAnalyzer: the pattern showed an optimum at 573 bp and the concentration was determined at 529 pg/μL. The library concentration equivalence was calculated as 1.69e^10^ molecules/μL and clonally amplified with 0.13, 0.25, 0.5 and 1 copies per bead (cpb) in 2 emPCR reactions per condition using the GS Titanium SV emPCR Kit (Lib-L) v2. The yields of the emPCR were respectively of 12.43, 15.48, 11.46 and 12.23 %, according to the expected quality of 5–20 % from the Roche procedure. The enriched clonal amplifications were loaded with 790,000 beads on the GS Titanium PicoTiterPlates PTP Kit 70x75 sequenced with the GS Titanium Sequencing Kit XLR70. The runs were performed overnight and were then analyzed on the cluster through the gsRunBrowser and gsAssembler_Roche. We obtained 349,885 total reads for *K. pneumoniae* subsp. *ozaenae* and 499,562 reads for *K. pneumoniae* subsp. *rhinoscleromatis*. The set of reads obtained from the two different sequencing methods were assembled with the Mira assembler v3.2. [16]. The resulting contigs were combined using Opera software v1.2 [17] in tandem with GapFiller V1.10 [18] to reduce the dataset. Finally, manual refinements were made using CLC Genomics software (CLC bio, Aarhus, Denmark) and homemade tools. These two newly-sequenced genomes were deposited at EMBL-EBI under accession number CDJH00000000 for *K. pneumoniae* subsp. *ozaenae* and CDOT00000000 for *K. pneumoniae* subsp. *rhinoscleromatis*. For the annotation process, assembled DNA sequences of the new draft genomes were run through various annotation applications including RNAmmer [19], Prodigal [20], ARAGORN [21], Rfam [22], Pfam [23], and Infernal [24].

### Genome sequence comparison and pan-genome analysis

We retrieved from NCBI the genome sequences of five strains of *K. pneumoniae* subsp. *pneumoniae* including *K. pneumoniae pneumoniae* HS11286 [Genbank: CP003200] [25], MGH 78578 [Genbank: CP000647] [26], 1084 [Genbank: CP003785] [27], NTUH-K2044 [Genbank: AP006725] [28], Ecl8 [Genbank: NZ_CANH00000000] [29], *K. pneumoniae* KCTC 2242 [Genbank: CP002910] [30] , two strains (E718 and KCTC 1686) of *Klebsiella oxytoca* [Genbank: CP003683 and CP003218, respectively][31, 32], *Klebsiella variicola* At-22 [Genbank: CP001891] [33] and *Klebsiella mobilis* EA1509E [Genbank: FO203355] (Table 1).

**Table 1:** General genome features

Species and Subspecies	Type strain	Status	Genome size (Mb)	GC content (%)	ORF	rRNA	tRNA	Genome accession no.	References
*Klebsiella pneumoniae pneumoniae*	HS11286	Complete	5.68	57.1	5,779	25	86	CP003200	Liu et al. (2012) [25]
*Klebsiella pneumoniae pneumoniae*	MGH 78578	Complete	5.69	57.2	5,184	25	85	CP000647	McClelland et al. (2006) [26]
*Klebsiella pneumoniae*	KCTC 2242	Complete	5.46	57.3	5,152	25	87	CP002910	Shin et al. (2012) [30]
*Klebsiella pneumoniae pneumoniae*	1084	Complete	5.39	57.4	4,962	25	79	CP003785	Lin et al. (2012) [27]
*Klebsiella pneumoniae pneumoniae*	NTUH-K2044	Complete	5.47	57.4	5,262	25	85	AP006725	Wu et al. (2009) [28]
*Klebsiella pneumoniae pneumoniae*	Ecl8	Complete (with gaps)	5.53	57.2	5,177	31	82	HF536482	Fookes et al. (2013) [29]
*Klebsiella pneumoniae pneumoniae ozaenae*	ATCC11296	Draft	4.95	57.5	4,818	3	62	CDJH0000000	Drancourt et al. (2001) [34]
*Klebsiella pneumoniae pneumoniae rhinoscleromatis*	Urmite	Draft	5.35	57.3	5,363	4	64	CDOT0000000	-
*Klebsiella variicola*	At–22	Complete	5.46	57.6	4,996	25	85	CP001891	Pinto-Tomas et al. (2009) [33]
*Klebsiella oxytoca*	E718	Complete	6.57	55.52	5,923	25	85	CP003683	Liao et al. (2012) [31]
*Klebsiella oxytoca*	KCTC 1628	Complete	5.98	56	5,340	25	85	CP00321	Shin et al. (2012) [32]
*Klebsiella mobilis*	EA1509E	Complete	5.59	54.93	5,117	26	88	FO203355	Diene et al. (2013) [2]

To functionally annotate protein sequences, we used the WebMGA function prediction workflow [35] and the NCBI COG database for prokaryotic proteins [36]. All hits below the default RPSBLAST e-value of 1e-03 were reported [37]. We performed a Principal Component Analysis (PCA) for all *K. pneumoniae* strains of the COG content using the R package (http://CRAN.R-project.org). We assigned KEGG orthology (KO) to the studied protein sequences using the KEGG automatic-annotation server (KAAS) [38] and mapped the KO-assigned genes to the Kyoto Encyclopedia of Genes and Genomes (KEGG) functional modules [39].

We determined the pan-genome composition of the six *K. pneumoniae* strains with and without including one of the other studied genomes *K. pneumoniae* subsp. *ozaenae* or *K. pneumoniae* subsp. *rhinoscleromatis* or *K. variicola* or *K. oxytoca.* Therefore, TBLASTN was performed to search the translated nucleotide database constituted of the different studied genomes using the proteomes as queries [37]. For each query, the query bit score was divided by the maximum bit score for all genomes in order to calculate the Blast Score Ratio (BSR) [40–43] allowing the conservation of peptides in each genome to be defined. Genes with a value of BSR ≥ 0.4 (equivalent to a ≥ 40 % protein identity over 100 % of the protein length) were considered to belong to core. This algorithm allows comparative analysis of multiple proteomes and nucleotide sequence to be performed simultaneously.

### Single Nucleotide Polymorphism (SNP) analysis

We identified SNPs among the core genomic regions using the Panseq package [1, 44, 45]. Multiple sequence alignments were built using MEGA 6.06 software [46] and phylogenies were reconstructed using the maximum likelihood method (PhyML) with 100 bootstrap iterations [47].

## Results

### Comparative genomic analysis of *Klebsiella pneumoniae* genomes

The final draft genome of *K. pneumoniae* subsp. *ozaenae* strain ATCC 11296 consists of 23 scaffolds [EMBL: LN681173-LN681195] and 128 contigs, containing 4,955,887 bp and a GC content of 57.5 %. For *K. pneumoniae* subsp. *rhinoscleromatis* strain Urmite, the draft genome consisted of 26 scaffolds [EMBL: LN776221-LN776246] and 135 contigs, containing 5,342,094 bp and with a GC content of 57.3 %. The major features of the *Klebsiella pneumoniae* sequenced genomes are summarized in Table 1.

All the studied *K. pneumoniae* genomes had an average length of 5.44 Mb. The *K. pneumoniae* subsp*. ozaenae* genome was the smallest with only 4.95 Mb and *K. pneumoniae* subsp. *pneumoniae* MGH 78578 was the largest genome with 5.69 Mb. The GC content varied from 57.1 % for *K. pneumoniae* subsp. *pneumoniae* HS11286 to 57.5 % for *K. pneumoniae* subsp. *ozaenae* with an average of 57.3 %. The number of predicted proteins in *Klebsiella pneumoniae* ranged from 4,818 for *K. pneumoniae* subsp. *ozaenae* to 5,779 for *K. pneumoniae* subsp. *pneumoniae* MGH 78578. A single ribosomal RNA operon (16S-23S-5S) was predicted for *K. pneumoniae* subsp*. ozaenae* and for the other strains, ranging from 8 to 9 operons. The number of tRNAs also differed depending on the species, ranging from 62 tRNA in *K. pneumoniae* subsp. *ozaenae* to 87 in *K. pneumoniae* KCTC 2242. The hierarchical clustering of the strains based on the number of tRNAs showed that *K. pneumoniae* subsp. *ozaenae* did not cluster with any other strains (Additional file 1). Altogether, *K. pneumoniae* subsp. *ozaenae* had the smallest genome size, number of genes, number of rRNAs and tRNAs among the *K. pneumoniae* strains. The reduced genome content suggests that *K. pneumoniae* subsp. *ozaenae* is more specialized than the other strains [48, 49]. Indeed, the evolution of specialized bacteria consists principally of gene loss [50], as investigated in particular for *Rickettsiales* [50, 51].

### Pan-genome and taxonomy

**Fig. 2:**
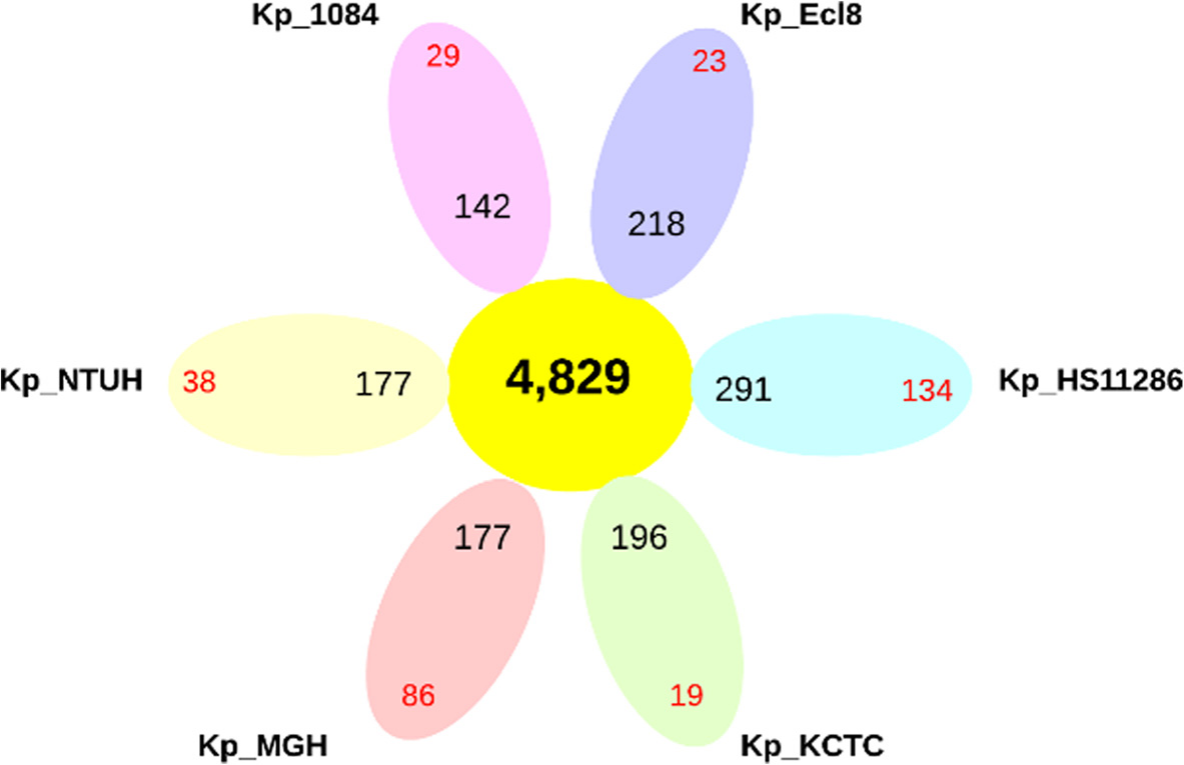
Pan-genome representation for 6 analysed strains of *Klebsiella pneumoniae.* The number of core genes is shows in the *yellow circle*. For each strain, the number of accessory genes is show in *black* and the number of unique genes is show in *red*

**Fig. 3:**
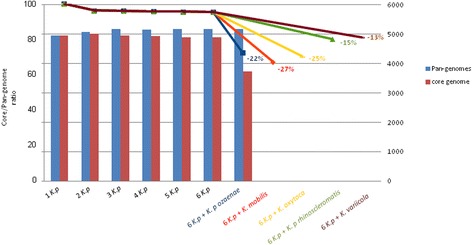
Distribution of the percentage of the core/pan-genome ratio (values on left y-axis) for all strains of *Klebsiella* and the pan-genome and the core genome (the values on right y-axis) for six strains of *Klebsiella pneumoniae*

The pan-genome for the six strains of *Klebsiella pneumoniae* contained 4,829 core genes (Fig. 2) and the core/pan-genome ratio was 94 %. This high percentage (more than 90 %) was indicative of a high rate of conservation among these strains [44]. When the different *Klebsiella* species were included, the core/pan-genome ratio decreased to 67 % with *K. mobilis*, 69 % with *K. oxytoca* and 81 % with *K. variicola* (Fig. 3). Altogether, a discontinuous variation of 13 to 27 % was observed between the *bona fide Klebsiella* species.

**Fig. 4:**
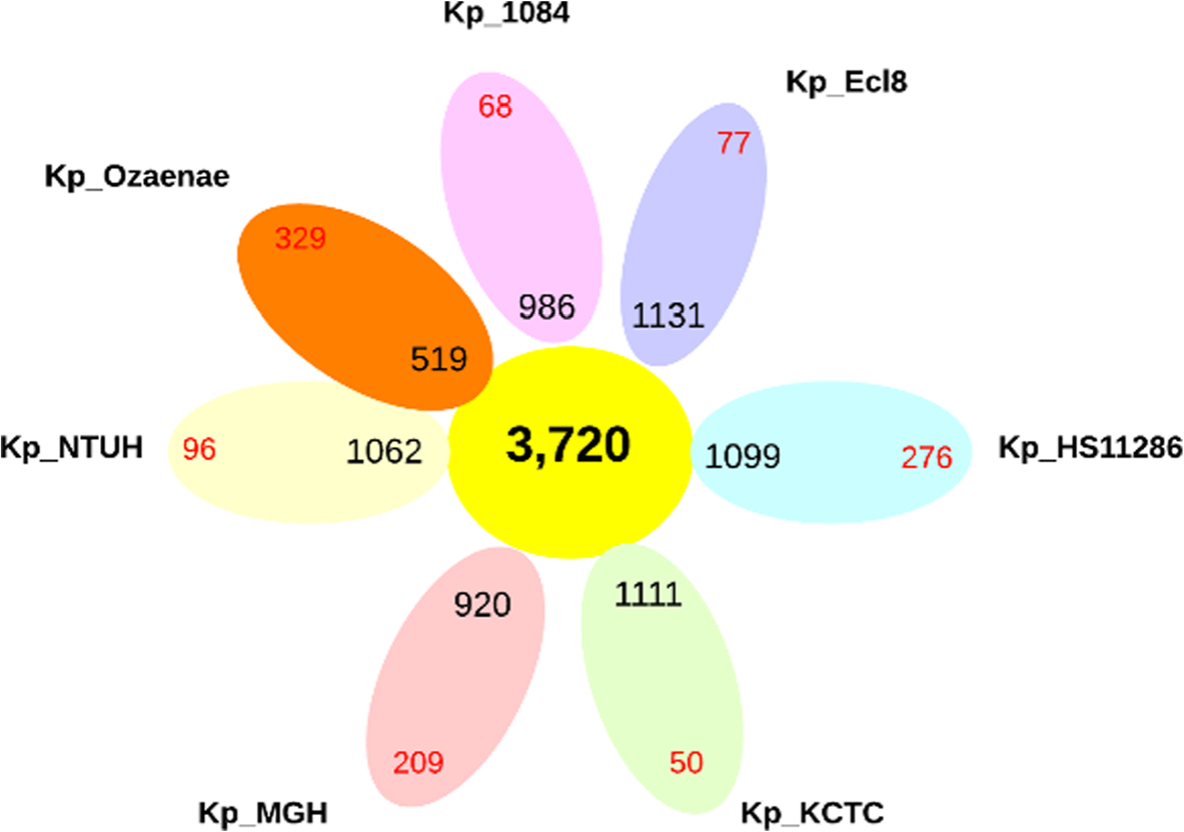
Pan-genome representation for 6 analysed strains of *Klebsiella pneumoniae* including *Klebsiella pneumoniae* subsp. *ozaenae.* The number of core genes is shows in the *yellow circle*. For each strain, the number of accessory genes is show in *black* and the number of unique genes is show in *red*

When *K. pneumoniae* subsp. r*hinoscleromatis* was included, the pan-genome expanded to 5,268 genes with 4,164 core genes. The core/pan-genome ratio was of 79 %, with a decrease of 15 % (Fig. 3). When *K. pneumoniae* subsp. o*zaenae* was included, the pan-genome expanded to 5,190 genes with 3,720 core genes (Fig. 4). The main differences between the core genes corresponded to genes with metabolic functions in starch and sucrose metabolism, galactose metabolism and citrate cycle. The core/pan-genome ratio was of 72 %, with a decrease of 22 % (Fig. 3). The rough decrease of the core/pan-genome ratio following the introduction of two strains of *K. pneumoniae* highlighted the very distinct genomic content of *K. pneumoniae* subsp. *rhinoscleromatis* and *K. pneumoniae* subsp. *ozaenae.* This discountinious variation was comparable to that previously observed among different species, supporting the claim that *K. pneumoniae* subsp. *rhinoscleromatis* and *K. pneumoniae* subsp. *ozaenae* are rather distinct species of *Klebsiella* than strains of *K. pneumoniae.*

### The specific genomic features of *K. pneumoniae* subsp. *ozaenae*

**Fig. 5:**
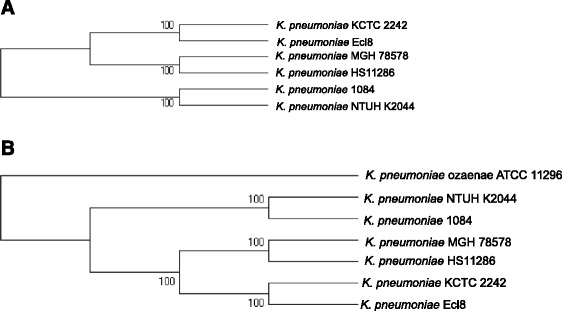
**a**: Single nucleotide polymorphisms of the core genes content based tree A. for the 6 strains of *Klebsiella pneumoniae* **b**. for the 6 strains of *Klebsiella pneumoniae* including *Klebsiella pneumoniae* subsp. *ozaenae*. These is a PhyML tree with 100 bootstrap iterations

The phylogenetic tree resulting from the SNPs of the core genome of the studied strains of *K. pneumoniae* showed a monophyletic group containing the *K. pneumoniae* subsp. p*neumoniae* (Fig. 5a) while *K. pneumoniae* subsp. *ozaenae* formed a distinct group (Fig. 5b). The analysis of the single nucleotide polymorphism along the core genome sequence presented *K. pneumoniae* subsp. *ozaenae* as a phylogenetically distinct entity within *Klebsiella,* that is distant from the other *K. pneumoniae* strains. Thus, the phylogenetic tree created based on SNPs of the core-genome showed that the genomic sequence of *K. pneumoniae* subsp. *ozaenae* is very different from that of the other *K. pneumonia* strains. Indeed, genome alignment of *K. pneumoniae* subsp. *ozaenae* with the six other strains of *K. pneumoniae* using MAUVE software [52] showed a large rearrangement of *K. pneumoniae* subsp. *ozaenae* with different inversion and deletions events (data not show). These findings strongly suggested the separation of *K. pneumoniae* subsp. *ozaenae* from the other *K. pneumoniae* strains and its recognition as a distinctive species.

When compared to the other strains of *K. pneumonia, K. pneumonia* subsp. *ozaenae* had fewer annotated proteins in all COG categories (4,572 proteins vs. 5,006 proteins on average) (Additional file 2). *K. pneumoniae* subsp. *ozaenae* lacked 202 genes (Additional file 3) that were present in all other *Klebsiella* strains and possessed 62 genes (Additional file 4) that were absent from all other strains. The missing genes from *K. pneumoniae* subsp. *ozaenae* encode for proteins involved in metabolism (13 %), information storage and processing (13 %) and cellular processes (8 %).

Likewise, the KO-annotation using KEGG server showed that *K. pneumoniae* subsp. *ozaenae* had fewer proteins (1,454) involved in metabolic pathways than the other *K. pneumoniae* strains (an average of 1,605 proteins), especially in amino acid metabolism, carbohydrate metabolim and xenobiotics biodegradation and metabolism. The analysis of the KEGG pathways for these genomes showed significant differences between *K. pneumoniae* subsp. *ozaenae* and the other *K. pneumoniae* strains in terms of their carbohydrate metabolism. The starch and sucrose metabolic pathways of *K. pneumoniae* subsp. *ozaenae* were deficient in the beta-xylosidase enzyme (EC:3.2.1.37) compared to the other *K. pneumoniae* strains.

Principal Component Analysis of the COG content, and hierarchical clustering calculated with the COG and KEGG data, respectively (Additional file 5, Fig. 6), showed that *K. pneumoniae* subsp. *ozaenae* did not cluster with any other *K. pneumoniae* strains. These findings suggest that *K. pneumoniae* subsp. *ozaenae* had differential functional content with specific pathways for carbohydrate metabolic in accordance with the phenotypic specificities observed *in vitro* for *K. pneumoniae* subsp. *ozaenae*.

**Fig. 6:**
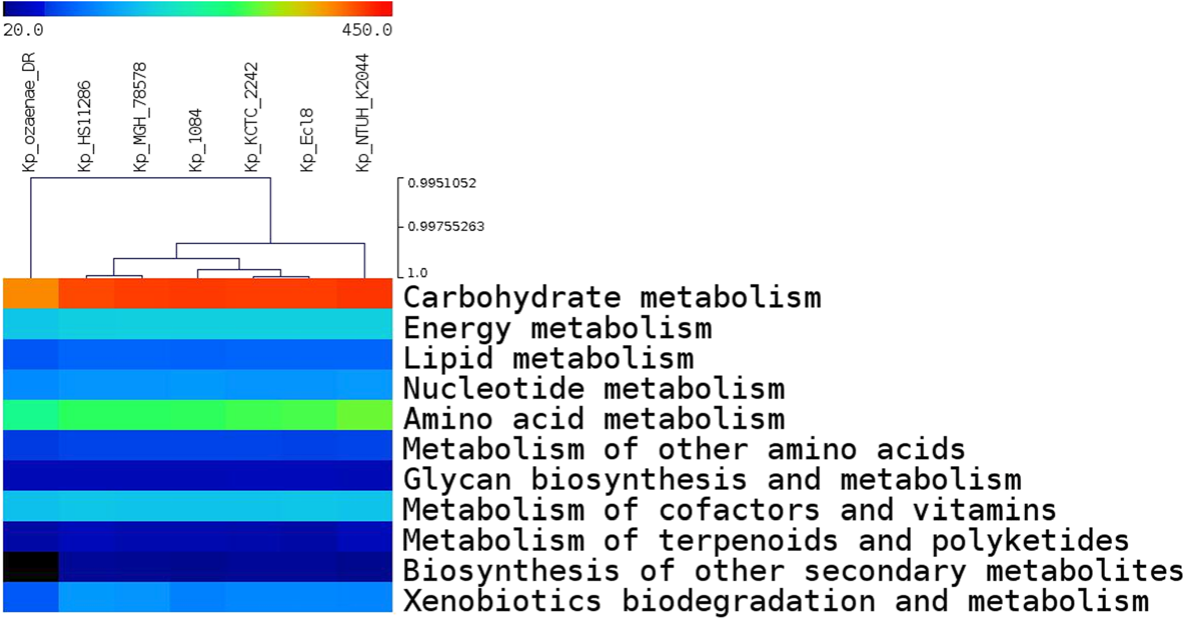
Hierarchical clustering of the *Klebsiella pneumoniae* strains based only on the KEGG distribution of the subclasses in the Metabolism category. The colors depend on the number of proteins implied in each metabolism category for each strain. The scale is represented in the figure

**Fig. 7:**
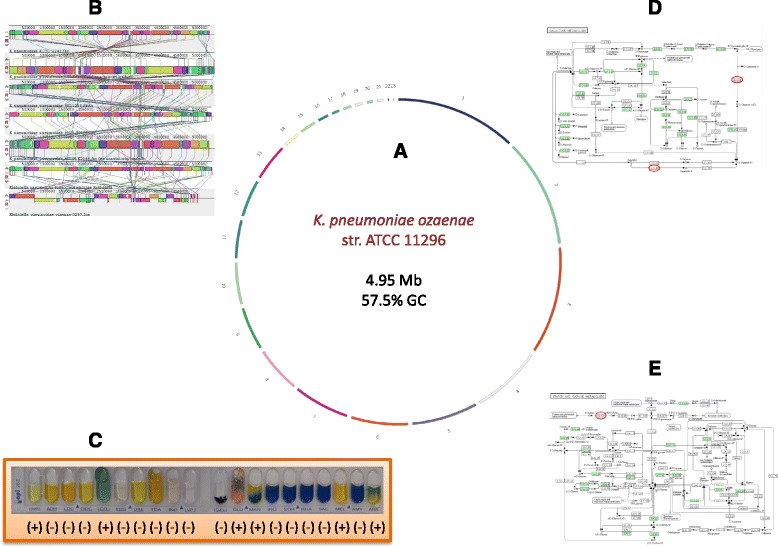
General representation of some distintive genomic and phenotypes of the *Klebsiella pneumoniae* subsp. o*zaenae* ATCC 11296 strain. **a**. Representation of the circular genome of *K.p.* subsp. o*zaenae* using Circos **b**. Genomes alignement of the 6 strains of *Klebsiella pneumoniae* including *Klebsiella pneumoniae* subsp. o*zaenae*. **c**. API20E identification of *Klebsiella pneumoniae* subsp. o*zaenae* ATCC 11296 strain. **e**. KEGG map of Galactose metabolism. **e**. KEGG map of Starch and Sucrose metabolism. The proteins surrounded in red are missing in *Klebsiella pneumoniae* subsp. o*zaenae*

We represented some genomic and phenotypic differences between *K. pneumoniae* subsp. *ozaenae* and other *Klebsiella pneumoniae* in the Fig. 7.

## Discussion

Bacterial taxonomy remains a complex and challenging field [53]. Initially, taxonomy was based on phenotypic criteria [5] related to a specific biological or medical interest. However, taxonomy has experienced a recent upheaval following the introduction of new genetic techniques. After the advent of DNA-DNA hybridization in 1979 [6, 53] many bacterial species were reclassified or removed from the taxonomic classification. More recently, the 16S rRNA gene has been used for the classification and nomenclature of bacterial species. This method often fails to reflect real distinctions between species [54]. The use of one single universal 16S rRNA gene can hardly be a realistic Tree of Life [54]. Furthermore, the accepted threshold of 1.3 % between two 16S rRNA sequences [9] required to differentiate between two different bacterial species seems to include almost 50 million years of the molecular clock [55, 56]. If we consider this threshold as the true species definition criterion, no bacterial lineages could have specialized in mammals [1, 57], which is an unacceptable conclusion. Because of the use of these criteria for the definition of bacterial species and the use of restrictive tools, the description of bacteria is very shallow and limited [58]. Bacteria with sympatric lifestyles, a high level of horizontal gene transfer [53, 59], large genomes, a significant number of ribosomal operons [60] and large pan-genomes [61, 62] compose bacterial species complexes. Only the isolation of a bacterium in a new niche or a significant population reduction will allow the appearance of a ‘specialist’, a *bona fide* species which will then present an allopatric lifestyle, a smaller genome, a reduced number of ribosomal operons and a smaller pan-genome [48].

We based our work on the hypothesis that the difference between two species exists as an irreconcilable difference. These species, thus, correspond to two distinct biological entities that could not be confused and could not transform into one another. A new nomenclature therefore needed to be introduced and pan-genomic studies are likely to be the most suitable method for exploring species under this system [44, 63]. Pan-genome study can identify different situations where speciation has occurred. First of all, an extremely broad continuum is defined as an infinite pan-genome, with a low core/pan-genome ratio. This indicates a lack of specialization in a bacterial group and the presence of a species complex or mixture that allows for the genesis of a species rather than a real species. In this context, *Shigella* can certainly be placed among *Escherichia coli* species [64]. Nevertheless, *Shigella* species are irreversibly different from *E. coli* species in terms of their metabolic, pathophysiological and genetic properties. *Shigella* spp. are human pathogens, *E. coli* complex clones, while *E. coli* strains are mostly commensals of the human intestine presenting a much larger genome repertoire [65].

In the context of *Klebsiella*, we began to define species using the pan-genome. The quantum discontinuous variation existing between the *Klebsiella pneumoniae* pan-genome and the other *Klebsiella* species shows that a discontinuous variation > = 10 % of the core/pan-genome ratio is observed by adding a single bacterial isolate. This major difference between genomes leads to a break in the ratio. This discontinuous variation corresponds to the start of a new mathematical function as previously described [44]. In a recent study, the best R^2^ (coefficient of determination) was determined in order to find the most accurate regression type. It has been shown that the addition of 9 *Shigella* strains to the 42 *E. coli* strains created a break in the core/pan-genome ratio and showed variation in their trend curve [44]. In quantum physics, such an abrupt change is similar to that of a discontinuous variation. Electrons revolve within discrete orbits. There is no gradual transition from one orbit to another; there are instead quantum discontinuous variations. This quantum phenomenon allows us to distinguish which transitions are progressive and which are quantic. The latter transition type results in the redefinition of species. The pan-genome study and calculation of the core/pan-genome ratio on the genomes of species that are theoretically the same should result in a linear graph. In practice however, we noticed a break event that prompted us to question the definition of a species. Differences between two species would necessarily be a striking phenomenon (ratio differences > 10 %) without a transition zone (Fig. 3) with irreconcilable differences. These physical phenomena fit well the definition of the species. This is not a shift that reflects the natural variability of species, but is instead a distinct biological phenomenon. According to this perspective, the criteria definition based on the species differentiation of *Klebsiella pneumoniae* enables us to show that *Klebsiella ozaenae* and *Klebsiella rhinoscleromatis*, which were initially believed to be individual species [4, 66] and were later considered to be sub-species of *Klebsiella pneumoniae* [67], are actually distinct biological entities that should indeed be considered as species. We believe that the emergence of a pan-genome will allow for the development of a more rational approach to species definition, in which species are defined as circumscribed and distinct biological entities with large differences that prevent them from transforming into closely-related species. We acknowledge the fact that pan-genome-based species classification may evolve with the discovery of new isolates. The definition of bacterial speciation, however, should reflect the restricted capacity of the species to obtain new characteristics and to adapt to any ecological changes.

## Conclusions

We have proposed a new tool for defining bacterial species using pan-genome analysis. This new method was applied to different species of the *Klebsiella* genus. We compared the core/pan-genome ratio of different species, which allowed us to take a great discontinuous variation forward in bacterial taxonomy. We found that *K. pneumoniae* subsp. *ozaenae* and *K. pneumoniae* subsp. *rhinoscleromatis* exhibit as many differences between them as those of *Klebsiella* genus, and demonstrated that these are distinct species of *Klebsiella* genus.

## Reviewers’ comments

*We thank the reviewers for their valuable comments and helpful suggestions. We would like to respond and revise our manuscript in light of the reviews.*

### Reviewer's report 1

*Prof. William Martin, Institut of Botanic III, Heinrich-Heine University, Düsseldorf, Germany*

### Reviewer 1

This is a very well written and interesting paper. I like it a lot. Few papers deal with species concepts among bacteria in such a relaxed and readable manner. Clearly, for clinical reasons we have to have species so that doctors can tell us what infection we have and how to treat it. Pragmatic approaches to the problem are useful, and this paper makes progress in that direction.line 83. "clear leap". In the vernacular of traditional systematics, this leap is called "discontinuous variation", so the principle has precedent. One might have a read of some classical systematic s papers for other kinds of organisms, following the keyword lead "discontinuous variation" in the lierature, and maybe rethink the title accordingly. Basically this paper suggests using a very traditional criterion with very modern data (pangenomes).

### Authors' response

*We thank Prof. Martin for his comments on our manuscript. We are pleased that you have enjoyed it. We replaced in this paper the word “leap” by “ discontinuous variation” acording to your advice.*

l. 111, define cpb

### Authors' response

*Cpb means copies per bead, we corrected this on line 108.*l. 167, Standard MCL clustering techniques could also be used here instead of blast score ratios.

### Authors' response

*The Blast Score Ratio is an algorithm that provides information concerning conserved genes between genomes (orthologs), it also shows their level of conservation (lines 146). The threshold used gives us an estimate of genetic variability. This is why we chose to use the BSR instead of standard MCL clustering.*

l. 261, worse than the clock issue is that rDNA does not clearly predict what the rest of the genome harbours, as pangenomes and this paper show.

### Authors' response

*Thank you for your comments.*

l. 271 "could not transform into one another" is not a very useful criterion because it makes untestable assumptions about what might happen in the future …

### Authors' response

*We mean that genomic content reflects the ecosystem. If the bacterium were to change its ecosystem and become specialized, no return would then be possible because no exchange is possible (lines 251, 292).*

l. 279, is "irreversibly" the right word here?

### Authors' response

*Yes, the word is “irreversibly”.*

l. 283, here we are getting to the main course of the apper. Maybe explain in more detail what Fig. 7 shows and perhaps find a mathematical decription for the dip ("discontinuous variation") in the c/p ratio that is independent of the value "10 %", which some might think is the sugestion for a pangenome defined species boundary, more studies on other species would be needed to get a better feel.

### Authors' response

*To clarify, we have reviewed many parts of this paper and discussed more about a mathematical description with an other example of a pan-genomic study, lines 273 to 277.*

l. 286, break — > discontinuity

### Authors' response

*Yes, “break” means “discontinuity”*

l. 287, nut orbitals are different, because sampling of further atoms will not uncover transitional orbitals, but sampleing of other srains will uncover transitional genomes, probably. But one gets the idea.

### Authors' response

*We have added another example of pan-genomic study performed in another study line 279.*

l. 296, which species definition? its a vast literature.

### Authors' response

*We gave a prokaryotic species definition on page 12. For more precision, we have added some references (43, 44, 49) on lines 236, 239, 243, 251.*

l. 302 f, what we see here is not a clear recommendation of the type that Stackebrandt would issue, but a pleas for the use of pangenomic data for the species question, which is unquestionalby reasonable and likely a fruitful avenue of pursuit.

### Authors' response

*Thank you for this comment.*

l. 314 … demonstrated that these are distinct species of Klebsiella genus at a level of

pangenomic discontinuity that would go undetected in a system rDN-based species definitions. Microbial systematics has always adapted to new technologies as it regards species boundaries, perhaps the next generation of adaptation is upon us now with the availability and utility of pangenomes, at least in the clinical context.

### Authors' response

*Thank you for this comment.*

In summary, this is a very fine paper, I enjoyed it a lot.Quality of written English: Acceptable

### Reviewer's report 2

*Dr. Pierre Pontarotti, Evolution Biologique et Modélisation, Aix-Marseille University, Marseille, France*

### Reviewer 2

The idea proposed in this article i.e. use of the complete genome comparison methodology to define biological species , is really interesting. Therefore, the concept deserves to be published.

However, in the present form the article is really difficult to understand. I recommend that it should be rewritten especially abstract, material and method , result and legend section to make them more precise and understandable.

### Authors' response

*We thank Dr. Pontarotti for his comments. We have rewritten parts of our manuscript as recommended.*

Concerning the discussion about the quantum leap, the author should discuss the possibility of intermediate species, that are not yet described. In other words, the quantum leap could be due to missing data.

The authors proposal remind me of punctual equilibrium theory from Elderdge and Gould which is based in part on fossil records. One of the argument against their theory was the possibility of missing fossils.

### Authors' response

*We acknowledge the fact that all Klebsiella species might not be yet known and therefore the discovery of future isolates may modify a little the proposed classification. We added this comment on the discussion (line 300).*

Quality of written English: Needs some language corrections before being published.

### Reviewer's report 3

*Dr. Pere Puigbo (nominated by an Editorial Board member, Dr Yuri Wolf), NCBI, NIH, Bethesda, USA*

### Reviewer 3

This article presents an interesting framework to handle the problem of defining (quantitatively) prokaryotic species. The authors use the simple, yet apparently efficient, core/pan-genome (C/P) ratio to define species of the genus Klebsiella. Overall, this ratio has the potential to be a useful tool to classify prokaryotic species. However, I think the article opens several technical and conceptual questions that may be addressed here.

- The authors tested the C/P ratio on Klebsiella species, but how it will perform in other species is still uncertain (e.g., intracellular parasites). Moreover, it would be very useful to see an example without a predefined group of closely related species to evaluate the real potential of this ratio in prokaryotic classification

### Authors' response

*We thank Dr. Puigbo for his comments concerning our manuscript. An identical study has been already performed on other species in our lab (ref. 34). I added these results to the discussion section line 277.*

- I think the “quantum leap” and the threshold identified in Klebsiella (>10 %) needs some randomization test and further exploration in other species. This introduces questions on how to use the C/P ratio: 1) is there any “golden threshold” that can be used across different taxonomical groups? 2) How is this threshold affected by genome reduction and horizontal gene transfer?

### Authors' response

*In a previous study, Rouli et al. (ref. 34), using a similar approach, observed that the C/P ratio varied from genus to genus and increased when genome size decreased. The influence of horizontal gene transfer on the C/P ratio, however, remains to be determined.*- The definition of species in page 3 is quite vague. It is improved on page 13, when the authors define the working hypothesis. However, I feel the article is missing a longer discussion about the meaning of ‘prokaryotic species’. It might be useful to expand this section and include additional references (e.g., PMID19411599, PMID21943000, PMID21714936)

### Authors' response

*We have clarified the definition on page 3, on discussion page 12 and we have added the 3 references mentioned.*

Quality of written English:

Acceptable

## Additional files

Additional file 1:
**Hierarchical clustering of the** ***Klebsiella pneumoniae*** **strains based on the number of aminoacil transfer RNAs.** Colors represented the number of proteins implied for each tRNA for each strain. The scale is included in the figure. (PNG 53 kb) Additional file 2:
**Number of genes for all species studied associated with the 25 general COG functional categories.** The Information storage and processing category is shown in red, the Cellular processes and signaling category is shown in green and the Metabolism category is shown in blue. The remaining items shown in white belong to the Poorly characterized category. A: RNA processing and modification; J: Translation, ribosomal structure and biogenesis; K: Transcription; L: Replication, recombination and repair; B: Chromatin structure and dynamics; D: Cell cycle control, cell division, chromosome partitioning; M: Cell wall/membrane/envelope biogenesis; N: Cell motility; O: Posttranslational modification, protein turnover, chaperones; P: Inorganic ion transport and metabolism; T: Signal transduction mechanisms; U: Intracellular trafficking, secretion, and vesicular transport; C: Energy production and conversion; Q: Secondary metabolites biosynthesis, transport and catabolism; E: Amino acid transport and metabolism; F: Nucleotide transport and metabolism; G: Carbohydrate transport and metabolism; H: Coenzyme transport and metabolism; I: Lipid transport and metabolism; R: General function prediction only; S: Function unknown. (PDF 27 kb) Additional file 3:
**Table showing the 202 genes, annotated by COG, present in the 6 strains of** ***Klebsiella pneumoniae*** **except** ***K. pneumoniae*** **subsp.** o*zaenae*. (TIFF 1443 kb) Additional file 4:
**Table showing 62 genes, annotated by COG, that are only present in** ***Klebsiella pneumoniae*** **subsp.** o*zaenae*. (TIFF 398 kb) Additional file 5:
**Plot of the Principal Component Analysis (PCA) axis of the COG content of the 6 strains of** ***Klebsiella pneumoniae*** **including** ***Klebsiella pneumoniae*** **subsp.** o*zaenae* using the R package. (TIFF 2723 kb)

## Abbreviations

SNP: Single nucleotide polymorphisms
ISP: Ion sphere particle
PGM: Personal genome machine
CPB: Copies per bead
CDSs: Coding DNA sequences
COG: Clusters of orthologs groups
MeV: Multiexperiment viewer
PCA: Principal component analysis
KEGG: Kyoto encyclopedia of genes and genomes
KO: KEGG orthology
KAAS: KEGG automatic annotation server
BSR: Blast score ratio
