# Supplementary figures and images for: Pan-genomic analysis to redefine species and subspecies based on quantum discontinuous variation: the Klebsiella paradigm
Source: Biol Direct. 2015 Sep 30;10:55. doi: 10.1186/s13062-015-0085-2 (PMC4588269; doi:10.1186/s13062-015-0085-2)

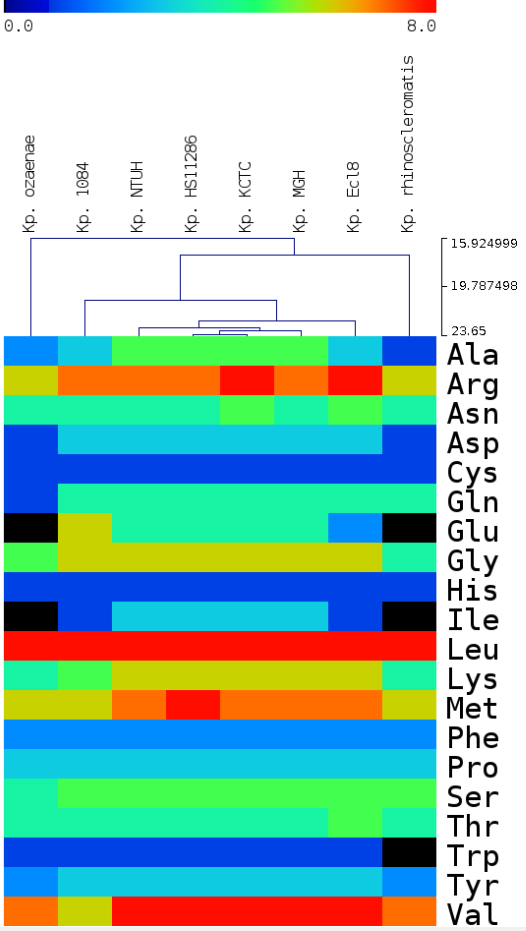

Supplement: Additional file 1: — Hierarchical clustering of the Klebsiella pneumoniae strains based on the number of aminoacil transfer RNAs. Colors represented the number of proteins implied for each tRNA for each strain. The scale is included in the figure. (PNG 53 kb) [file 13062_2015_85_MOESM1_ESM.png]

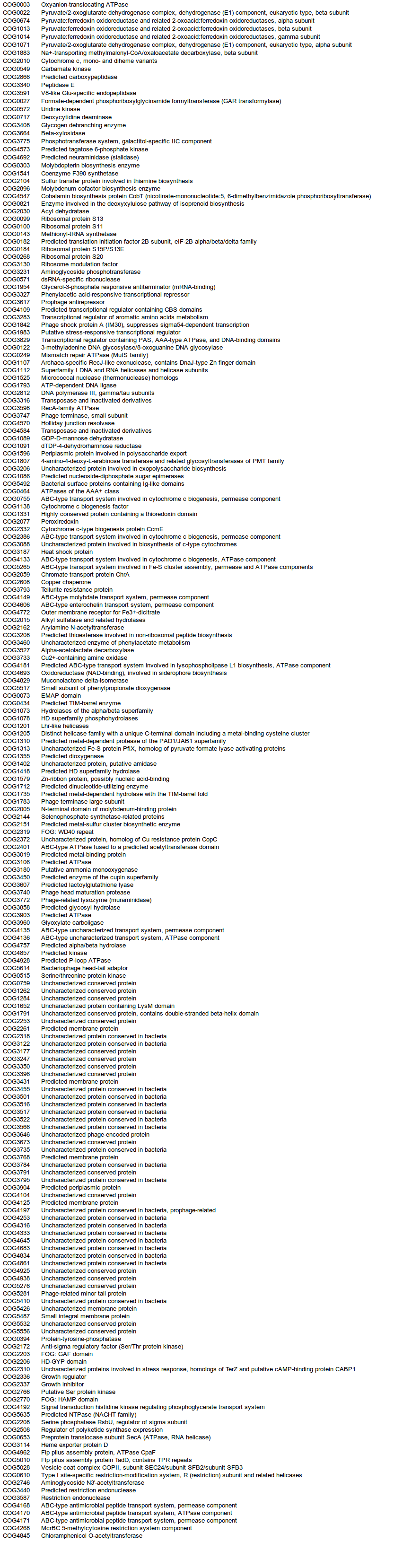

Supplement: Additional file 3: — Table showing the 202 genes, annotated by COG, present in the 6 strains of Klebsiella pneumoniae except K. pneumoniae subsp. ozaenae. (TIFF 1443 kb) [file 13062_2015_85_MOESM3_ESM.tiff]

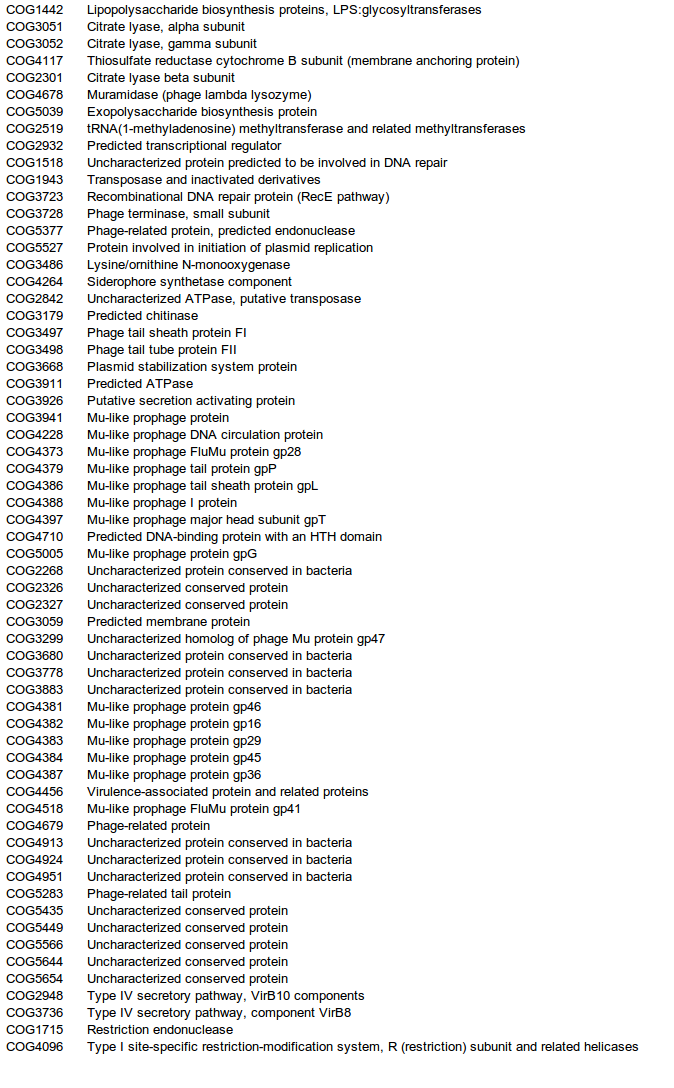

Supplement: Additional file 4: — Table showing 62 genes, annotated by COG, that are only present in Klebsiella pneumoniae subsp. ozaenae. (TIFF 398 kb) [file 13062_2015_85_MOESM4_ESM.tiff]

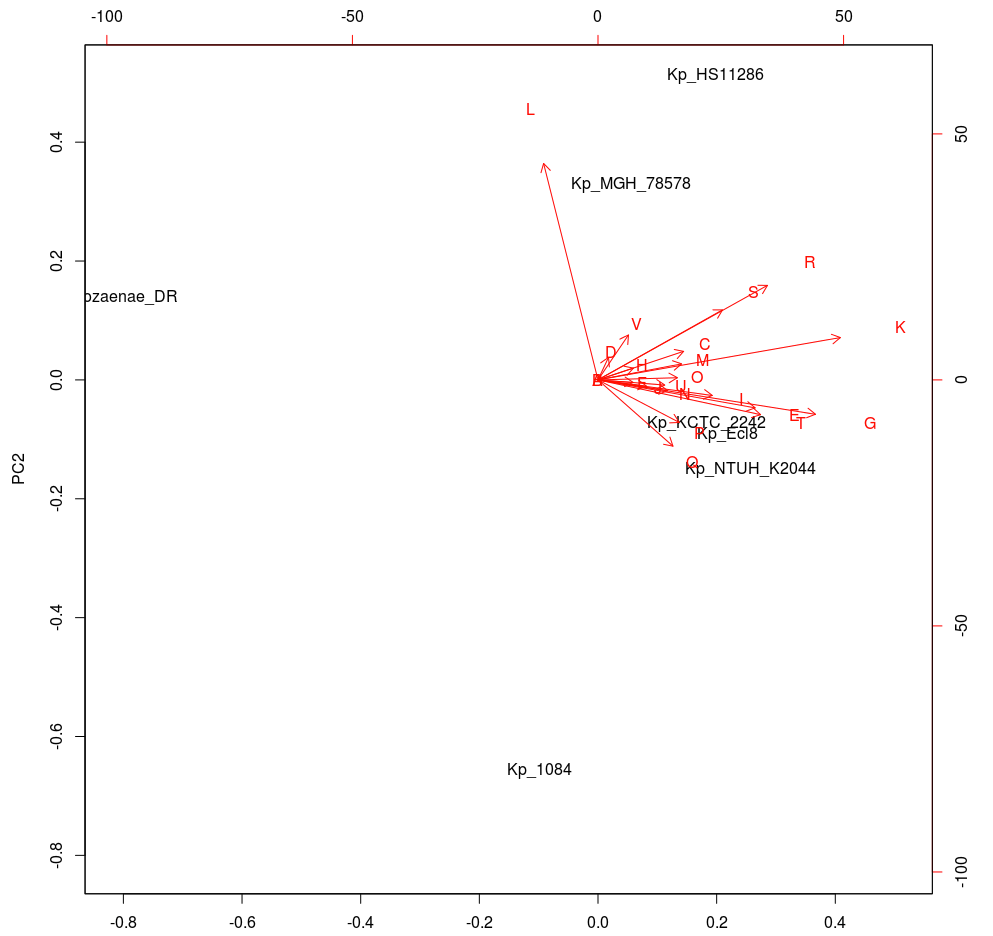

Supplement: Additional file 5: — Plot of the Principal Component Analysis (PCA) axis of the COG content of the 6 strains of Klebsiella pneumoniae including Klebsiella pneumoniae subsp. ozaenae using the R package. (TIFF 2723 kb) [file 13062_2015_85_MOESM5_ESM.tiff]
