# Supplementary material for: Pan-genomic analysis to redefine species and subspecies based on quantum discontinuous variation: the Klebsiella paradigm
Source: Biol Direct. 2015 Sep 30;10:55. doi: 10.1186/s13062-015-0085-2 (PMC4588269; doi:10.1186/s13062-015-0085-2)
Supplement: Additional file 2: — Number of genes for all species studied associated with the 25 general COG functional categories. The Information storage and processing category is shown in red, the Cellular processes and signaling category is shown in green and the Metabolism category is shown in blue. The remaining items shown in white belong to the Poorly characterized category. A: RNA processing and modification; J: Translation, ribosomal structure and biogenesis; K: Transcription; L: Replication, recombination and repair; B: Chromatin structure and dynamics; D: Cell cycle control, cell division, chromosome partitioning; M: Cell wall/membrane/envelope biogenesis; N: Cell motility; O: Posttranslational modification, protein turnover, chaperones; P: Inorganic ion transport and metabolism; T: Signal transduction mechanisms; U: Intracellular trafficking, secretion, and vesicular transport; C: Energy production and conversion; Q: Secondary metabolites biosynthesis, transport and catabolism; E: Amino acid transport and metabolism; F: Nucleotide transport and metabolism; G: Carbohydrate transport and metabolism; H: Coenzyme transport and metabolism; I: Lipid transport and metabolism; R: General function prediction only; S: Function unknown. (PDF 27 kb) [file 13062_2015_85_MOESM2_ESM.pdf]

Categories	*K.pneumoniae* 1084	*K.pneumoniae* Ecl8	*K.pneumoniae* HS11286	*K.pneumoniae* KCTC 2242	*K.pneumoniae* MGH 78578	*K.pneumoniae* NTUH K2044	*K.pneumoniae ozaenae* ATCC 11296
J	198	204	199	204	202	206	191
A	1	1	1	1	1	1	1
K	430	459	456	459	459	457	396
L	173	192	228	195	233	201	224
B	1	1	1	1	1	1	1
D	41	44	50	44	44	44	43
V	63	67	76	65	75	62	60
T	215	232	214	232	223	234	186
M	237	241	243	242	251	246	222
N	62	63	58	63	70	64	44
U	116	114	116	114	121	121	99
O	154	161	161	160	156	158	139
C	309	311	325	315	314	315	294
G	571	546	573	544	565	581	503
E	482	519	489	516	486	500	465
F	106	104	110	103	102	109	97
H	201	200	207	199	204	206	194
I	131	135	134	134	129	129	102
P	328	346	323	345	329	348	320
Q	133	116	121	114	110	125	97
R	511	553	564	546	526	541	508
S	392	417	426	417	409	410	386
Total	4855	5026	5075	5013	5010	5059	4572
